# Significance of Metformin Use in Diabetic Kidney Disease

**DOI:** 10.3390/ijms21124239

**Published:** 2020-06-14

**Authors:** Daiji Kawanami, Yuichi Takashi, Makito Tanabe

**Affiliations:** Department of Endocrinology and Diabetes Mellitus, Fukuoka University School of Medicine, Fukuoka 814-0180, Japan; y.takashi.si@fukuoka-u.ac.jp (Y.T.); mtanabe@live.jp (M.T.)

**Keywords:** metformin, diabetic nephropathy, diabetic kidney disease, CKD, cardiovascular disease

## Abstract

Metformin is a glucose-lowering agent that is used as a first-line therapy for type 2 diabetes (T2D). Based on its various pharmacologic actions, the renoprotective effects of metformin have been extensively studied. A series of experimental studies demonstrated that metformin attenuates diabetic kidney disease (DKD) by suppressing renal inflammation, oxidative stress and fibrosis. In clinical studies, metformin use has been shown to be associated with reduced rates of mortality, cardiovascular disease and progression to end-stage renal disease (ESRD) in T2D patients with chronic kidney disease (CKD). However, metformin should be administered with caution to patients with CKD because it may increase the risk of lactic acidosis. In this review article, we summarize our current understanding of the safety and efficacy of metformin for DKD.

## 1. Introduction

*Galega officinalis* is a perennial cold-resistant plant rich in guanidine [1]. Originally cultivated as a horticultural plant, it began to be used as herbal therapy for treating polyuria associated with diabetes in medieval Europe [2]. In 1918, guanidine was discovered to have hypoglycemic action [3]. Metformin is a biguanide derivative developed as the fusion of two guanidines. In 1998, the United Kingdom Prospective Diabetes Study (UKPDS) 34 demonstrated the safety and efficacy of metformin in obese patients with type 2 diabetes (T2D) [4]. Accordingly, metformin use has been shown to be associated with a reduced risk of micro- and macro-vascular complications in T2D patients in UKPDS80, a 10-year follow up of the post-trial monitoring [5]. These findings established the role of metformin in T2D treatment, particularly with regard to attenuating diabetic complications. It is now commonly accepted that metformin is an important therapeutic option as a first-line therapy for T2D worldwide. 

Diabetic kidney disease (DKD) is a leading cause of end-stage renal disease (ESRD). The inhibition of the onset and progression of DKD is an urgent issue; however, no treatment approach specific to DKD has yet been established. Therefore, anti-diabetic agents with renoprotection are awaited. 

A series of experimental studies revealed that metformin exerts renoprotective effects via multiple mechanisms. These beneficial effects can be expected clinically; however, the use of metformin should be determined depending on the renal function. In incipient DKD, metformin can be actively used, but its administration is not recommended in patients with advanced renal impairment because it may increase the risk of lactic acidosis. However, the potential efficacy of metformin on reducing the cardiovascular disease (CVD) risk in T2D patients with moderate chronic kidney disease (CKD) has also been suggested. 

In the present review article, we discuss our current understanding of the benefits of metformin use in DKD from both a basic and clinical standpoint.

## 2. Pathogenesis and Clinical Features of DKD

DKD is characterized as glomerulosclerosis and tubulo-interstitial fibrosis. Glomerular hyperfiltration, inflammation, oxidative stress and altered lipid metabolism have been implicated in the pathogenesis of DKD [6,7,8]. In addition, the release of cytokines and chemokines and infiltration of immune cells contribute to the onset and progression of DKD [9]. Inflammatory signaling pathways promote the mesangial expression of transforming growth factor (TGF)-β and production of extracellular matrix (ECM) via nuclear factor (NF)-κB activation [10], leading to the development of thickening of the glomerular basement membrane (GBM) and glomerulosclerosis [11]. These inflammatory and fibrotic responses also cause tubulo-interstitial fibrosis. Podocyte injury is also a key feature in the early stage of DKD. Podocytes participate in the formation of the filtration barrier and regulate glomerular filtration, along with the GBM and endothelium [12]. Therefore, podocyte loss under diabetic conditions results in the damage and hyperpermeability of glomerular endothelial cells, leading to the development of albuminuria [13].

The assessment of both albuminuria and the estimated glomerular filtration rate (eGFR) decline is recommended for the clinical diagnosis of DKD [14]. In the traditional setting, albuminuria has been characterized as a clinical feature of DKD. It is considered that microalbuminuria later progresses to macroalbuminuria/proteinuria, which in turn precedes the GFR decline. However, heterogeneity of DKD has emerged. Recent epidemiological studies have shown the reduced prevalence of albuminuria in patients with DKD, whereas the prevalence of nonalbuminuric DKD is increasing [15,16,17]. Although the reason for these observations remains unclear, it has been proposed that non-albuminuric DKD may comprise individuals with an aging-associated reduced kidney function and those who responded well to renin-angiotensin system blockade [18]. 

Differences in the prognosis among DKD phenotypes are controversial. In the Renal Insufficiency and Cardiovascular Events (RIACE) Italian multicenter study, 15,773 Italian T2D patients with an eGFR < 60 mL/min/1.73 m^2^ were enrolled. This observational study demonstrated that the mortality risk associated with a reduced eGFR alone was similar to that associated with albuminuria alone during median follow up of 7.4 years [19]. A post-hoc analysis of the Action in Diabetes and Vascular disease: preterAx and diamicroN-MR Controlled Evaluation (ADVANCE) including 10,640 T2D patients showed that the risk of CV death in non-albuminuric DKD individuals are not higher than those in albuminuric DKD individuals [20]. Furthermore, another observational cohort study including about 3000 Japanese T2D patients (median follow-up: 9.7 years) demonstrated that non-albuminuric DKD is not associated with an increased risk of mortality, CVD, or a renal function decline compared with no-DKD and albuminuric DKD individuals with or without reduced eGFR (<60 mL/min/1.73 m^2^) [21]. These findings indicate that both albuminuria and eGFR decline are important therapeutic targets in DKD.

Histological differences in these DKD phenotypes have been reported. Non-albuminuric DKD has been hypothesized to be associated with atypical vascular and/or tubulo-interstitial lesions, instead of the typical glomerular lesions [16]. Ekinci et al. found that the renal biopsy samples of 31 T2D patients with a reduced eGFR (<60 mL/min/1.73 m^2^) showed typical glomerular lesions of DKD among the patients with albuminuria [22]. In contrast, these changes were seen less frequently in those patients without albuminuria, likely reflecting the greater contributions of aging, hypertension and arteriosclerosis [22]. Furthermore, Shimizu et al. investigated 260 T2D patients with biopsy-proven DKD and found that glomerular lesions were associated with albuminuria, whereas glomerular, tubulo-interstitial and vascular lesions were associated with a reduced eGFR [23]. Of note, specific histological changes in DKD phenotypes cannot be determined because the wide heterogeneity of renal lesions has been reported in T2D with albuminuria [24]. Most anti-diabetic agents, including biguanides, thiazolidinediones, sodium glucose cotransporter (SGLT) 2 inhibitors, dipeptidyl peptidase (DPP)-4 inhibitors and glucagon-like peptide (GLP)-1 receptor agonists (GLP-1RAs), have been shown to exert renoprotective effects with different evidence levels [25] in glucose lowering-dependent and glucose lowering-independent mechanisms [7,26]. Clinically, intensified multifactorial intervention, including that for hyperglycemia, hypertension and dyslipidemia, has been shown to attenuate DKD in patients with T2D [27]. As will be described later, metformin is considered to have favorable effects on renal inflammation, oxidative stress and fibrosis under diabetic conditions. Therefore, metformin potentially exerts renoprotective effects irrespective of the DKD phenotype. In this review article, we will treat DKD as synonymous with diabetic nephropathy. However, cases in which whether or not the cause of CKD is diabetic is unclear will be described as T2D with CKD but not DKD.

## 3. Glucose-Lowering Mechanisms of Metformin

Metformin is proposed to inhibit hepatic gluconeogenesis in an AMP-activated kinase (AMPK)-dependent and independent manner. It enters hepatocytes through organic cationic transporter (OCT) 1, as shown by a study demonstrating a reduced metformin uptake in hepatocytes of OCT1-deficient mice [28]. In addition, genetic polymorphisms of OCT1 in humans that determine responses to metformin have been reported [29]. Metformin is excreted via the urine, which is mediated by renal OCT1 and OCT2 on the basolateral membrane of proximal tubule cells and multidrug and toxin extrusion (MATE) 1 on the apical membrane [30,31,32].

Metformin that entered the cells inhibits mitochondrial respiratory complex I, resulting in a reduction of ATP synthesis and an increase in the AMP/ATP and ADP/ATP ratios, leading to the phosphorylation of AMPK [33]. Liver kinase (LK) B-1 is required for AMPK phosphorylation and acts as a kinase upstream of AMPK [34]. LKB-1/AMPK plays an important role in inhibiting cAMP response element binding protein (CREB)-regulated transcription coactivator 2 (CRTC2), which enhances the transcriptional activation of the gluconeogenic genes. CRTC2 promotes CREB-mediated PPARγ coactivator (PGC)-1α transcription and its target genes, phosphoenolpyruvate carboxykinase (PEPCK) and glucose-6-phosphatase (G6Pase), key enzymes in gluconeogenesis [35,36,37]. Importantly, metformin has been shown to inhibit hepatic gluconeogenesis in LKB1- and AMPK-deficient hepatocytes by decreasing hepatic energy state [36], indicating that metformin also reduces hepatic gluconeogenesis in an AMPK-independent manner. In this regard, metformin has been shown to suppress gluconeogenesis by modifying the cellular redox state. Madiraju et al. demonstrated that metformin reduces gluconeogenesis by inhibiting mitochondrial glycerophosphate dehydrogenase, a redox shuttle enzyme [38]. They also showed that metformin inhibits hepatic glucose production in a redox state-dependent manner without altering the activity of acetyl-CoA carboxylase (ACC), a target of AMPK, or the gluconeogenic enzyme expression [39]. These findings support the notion that metformin inhibits hepatic gluconeogenesis in both an AMPK-dependent and AMPK-independent fashion.

**Figure 1:**
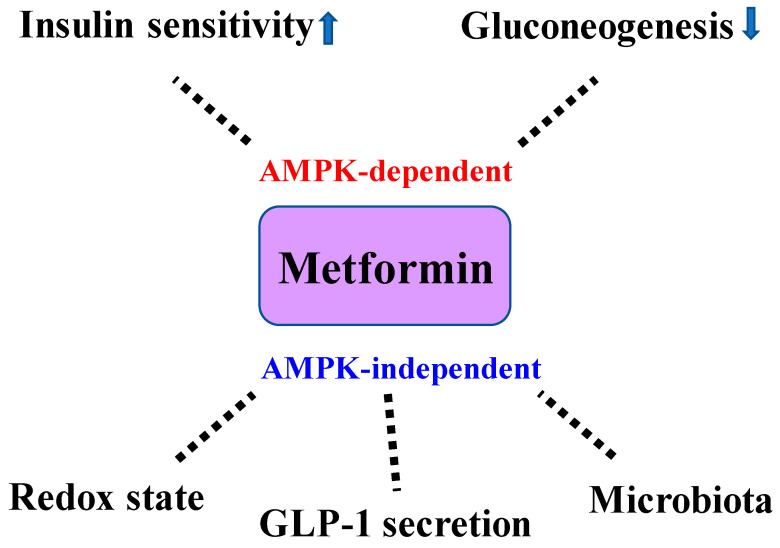
Glucose-lowering mechanisms of metformin. Metformin inhibits hepatic gluconeogenesis and improves insulin sensitivity via AMPK activation. Importantly, metformin has been shown to inhibit hepatic gluconeogenesis in the absence of AMPK. Changes in redox state and gut microbiota as well as GLP-1 secretion by metformin have been implicated in AMPK-independent glucose-lowering mechanisms. AMPK: AMP-activated kinase, GLP-1: glucagon-like peptide 1.

Changes in the gut microbiome induced by metformin may be involved in the improvement of the glucose metabolism [40]. In the present study, T2D subjects were randomly allocated to a placebo group or metformin group. At 4 months after metformin administration, fecal samples derived from the individuals with metformin use were transferred to germ-free mice. Interestingly, these mice showed an impaired glucose tolerance, which may have been mediated by *Bifidobacterium adolescentis* [40]. It is suggested that metformin increases GLP-1 secretion from the intestine by inhibiting intestinal absorption of bile acids [41]. Metformin’s ability to reduce intestinal glucose absorption may be involved in the increase in GLP-1 secretion in T2D patients [42]. Furthermore, it has been reported that metformin suppresses food intake and promotes weight loss via growth differentiating factor (GDF) 15 [43]. These effects may have contributed to the effects of metformin on glucose metabolism. Finally, metformin has been implicated in improving insulin sensitivity by increasing the insulin receptor tyrosine kinase activity and the recruitment and activity of GLUT4 glucose transporters in skeletal muscle cells [44]. The glucose-lowering mechanisms of metformin are shown in Figure 1.

## 4. Basic Mechanisms Underlying the Effects of Metformin on DKD

### 4.1. Glomerulosclerosis

The disturbance of the mesangial cell function plays an important role in the development of glomerulosclerosis under diabetic conditions [6]. Metformin has been shown to attenuate albuminuria by inhibiting the renal expression levels of TGF-β and ECM production, such as connective tissue growth factor (CTGF) in diabetic rats by inhibiting oxidative stress, inflammation and improving glucose and lipid metabolism [45]. Metformin has also been shown to attenuate high glucose-induced NF-κB activation and subsequent monocyte chemoattractant protein (MCP)-1 in rat mesangial cells [46]. Intriguingly, those favorable effects were mediated by the metformin-induced upregulation of GLP-1R. It has been reported that GLP-1R expression in the renal cortex is reduced in db/db mice [46]. Kim et al. demonstrated that lipotoxicity-induced apoptosis is mediated by GLP-1R downregulation in mesangial cells, which are prevented by metformin [47]. They also observed that diminished glomerular GLP-1R is restored by metformin in db/db mice [47]. Although the mechanisms by which metformin increases the GLP-1R expression remain unclear, it seems that AMPK is involved in this observation because both metformin and 5-amino-4-imidazolecarboxamide riboside (AICAR), an AMPK activator, induce the GLP-1R expression [48]. Taken together, these findings indicated that the combination of metformin and incretin-based therapy is effective for treating DKD. AMPK-dependent renoprotection by metformin has also been shown in a rat subtotal nephrectomy model of CKD. Borges et al. showed that 120-day administration of metformin reduces albuminuria and interstitial fibrosis by AMPK activation and subsequent improvement of mitochondrial biogenesis, all of which are mediated independent of the blood pressure and glucose reduction [49]. Long non-coding RNAs (lncRNAs) are a class of RNA molecules with a length of more than 200 nt that do not encode proteins [50]. It has been demonstrated that metformin represses proliferation, inflammation and ECM accumulation in mesangial cells by inhibiting the expression of H19, which is an lncRNA that upregulates the TGF-β expression [51]. AMPK may be involved in the metformin-induced modulation of lncRNA because metformin has been shown to inhibit endothelial cell proliferation and atherosclerosis by attenuating the lncRNA TUG1 by activating the AMPK/mammalian target of rapamycin (mTOR) pathway [52]. Metformin has been shown to modulate cytoskeleton dynamics and insulin sensitivity by AMPK activation in podocytes [53,54]. Furthermore, it has been shown to prevent albuminuria and podocyte apoptosis via the downregulation of oxidative stress and podocyte loss in T2D rats [55,56]. Lipid phosphatase Src homology 2 domain-containing inositol-5-phosphatase 2 (SHIP2) in the kidney is upregulated in diabetic mice as well as T2D patients and induces podocyte apoptosis by reducing insulin signaling and Akt activity [57]. The administration of metformin in db/db mice resulted in reduced podocyte apoptosis by reducing the SHIP2 activity [57]. Interestingly, the authors found that glomerular SHIP2 activity is not upregulated in metformin-treated T2D patients [57].

### 4.2. Tubular Injury/Renal Fibrosis

Under diabetic conditions, mTOR is activated and plays an important role in the damage, apoptosis and fibrotic response of renal cells as well as epithelial-to-mesenchymal transition (EMT) [58,59]. Metformin attenuates mTOR-mediated tubular injury under diabetic conditions [60,61,62]. LKB-1 and AMPK have been shown to prevent tubulo-interstitial fibrosis. Impaired fatty acid oxidation (FAO) in proximal tubular cells has been shown to be associated with TIF because of a reduced energy deficiency, which is prevented by metformin [63,64]. Metformin has been shown to attenuate tubulo-interstitial fibrosis via the phosphorylation of AMPK and its target ACC, which is a key regulator of FAO, thereby increasing the lipid availability [65]. Finally, metformin attenuates apoptosis by inhibiting advanced glycation end product (AGE)-mediated NF-κB activation and reactive oxidative species (ROS) generation in renal tubular cells [66,67]. These findings indicate that metformin has protective effects on glomerular constituent cells and renal tubular cells under diabetic conditions. 

A hypoxic condition has been implicated in the pathogenesis of DKD [68]. Metformin has been shown to be involved in oxygen metabolism under diabetic conditions. Takiyama et al. demonstrated that metformin inhibits HIF-1α, a central regulator of the hypoxia-mediated cellular response in proximal tubule cells [62]. They also found that metformin reduced the ATP production and oxygen consumption rates and increased cellular oxygen tension in T2D rats [62]. Christensen et al. showed that metformin improves medullary hypoxia and attenuates mitochondrial superoxide radical production by inhibiting uncoupling protein-(UCP) 2 under diabetic conditions [69]. Senescence of renal cells has been implicated in the pathogenesis of DKD [70]. Metformin has been shown to inhibit high glucose-induced expression of the senescence-associated gene p21 in renal tubular epithelial cells [71]. Furthermore, the administration of metformin in db/db mice has been shown to inhibit the senescence of renal tubular epithelial cells by increasing the RNA-binding protein muscle-blind-like splicing regulator (MBNL) 1 and miR-130a-3p expression and reducing the STAT3 expression [71].

Of note, metformin can attenuate tubulo-interstitial damage independent of OCTs and AMPK. As mentioned previously, OCT1 plays an important role in the metformin uptake by hepatocytes. However, Christensen et al. showed that administration of metformin attenuated unilateral ureteral obstruction (UUO)-mediated TNF-α, MCP-1 and the proximal tubule injury marker KIM-1 inductions in the kidney of OCT1/2-deficient mice [72]. They observed that metformin attenuates these inductions by UUO in AMPK-β1-deficient mice, suggesting that the renoprotective effects of metformin are independent of OCT1/2 and AMPK [72]. Accordingly, they reported that metformin inhibits STAT3-mediated immune cell infiltration, tubular damage and fibrosis in a UUO mice model, which may explain the AMPK-independent mechanisms [73]. Feng et al. showed that metformin attenuates UUO-induced renal fibrosis in AMPKα2-deficient mice [74]. They observed that metformin inhibits the TGF-β1 expression in an AMPKα2-dependent manner. By contrast, it inhibited TGF-β1 downstream Smad3 phosphorylation in an AMPKα2-independent manner [74]. These findings indicate that renoprotection by metformin occurs in both AMPK-dependent and independent manners. 

### 4.3. Autophagy

Autophagy is a protective mechanism for DKD and is regulated by AMPK and silent mating type information regulation 2 homolog 1 (Sirt1). Sirt1 is a NAD^+^-dependent deacetylase and increases the expression of FoxO1, a transcription factor that can reduce oxygen-free radicals by inducing autophagy [75,76]. Therefore, the AMPK and Sirt1/FoxO1 axis has been suggested to be a protective signaling pathway in autophagy. Metformin has been shown to attenuate renal fibrosis and histological changes in the glomerulus via autophagy by activating the AMPK/Sirt1/FoxO1 signaling pathway [76,77].

### 4.4. Urinary Sodium Excretion

Finally, the relationship between metformin and diabetes-related risk factors has been reported. Urinary sodium excretion is involved in regulating the blood pressure. Hashimoto et al. showed that sodium excretion is increased by metformin via the reduction in Na-Cl cotransporter (NCC) activity in the distal convoluted tubule [78]. Hyperuricemia is an independent risk factor for CKD in individuals with a normal kidney function in both the general population and subjects with diabetes [79]. Zhang et al. demonstrated that urinary metformin excretion is increased in hyperuricemic rats [30]. From a mechanistic standpoint, uric acid upregulates the expression of renal metformin transporters OCT1, OCT2 and MATE1, thereby promoting metformin excretion into the urine [30]. 

**Figure 2:**
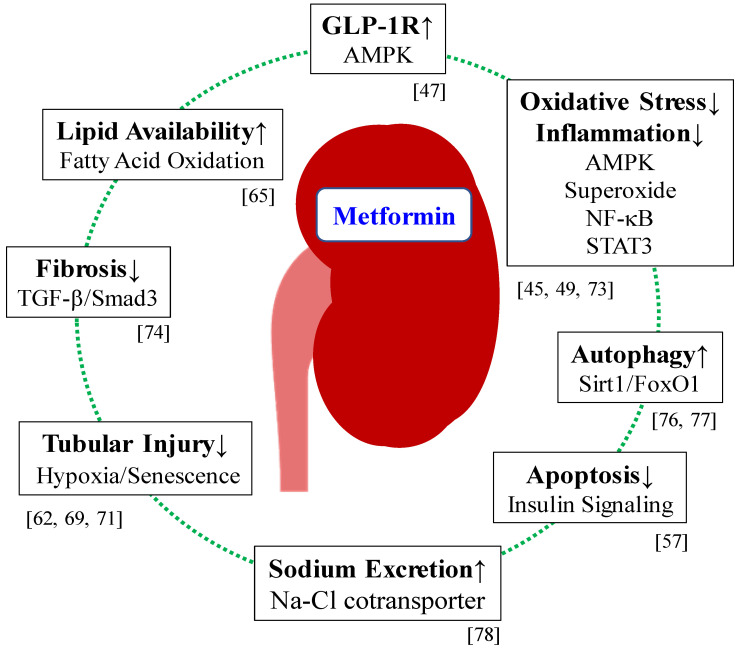
Renoprotective mechanisms of metformin. Metformin attenuates DKD in glucose lowering-dependent and glucose lowering-independent manners. AMPK plays an important role in the glucose-lowering effects as well as the pleiotropic effects of metformin. Metformin reduces the body weight (e.g., via GDF15 and GLP-1) and improves insulin resistance, activities that may underlie the beneficial effects of metformin on DKD. DKD: diabetic kidney disease, AMPK: AMP-activated kinase, GDF15: growth differentiating factor 15, GLP-1: glucagon-like peptide 1, GLP-1R: glucagon-like peptide 1 receptor.

Taken together, these findings suggest that renoprotection by metformin is mediated by attenuating oxidative stress, inflammation and fibrosis and inducing autophagy. Furthermore, metformin exerts renoprotective effects in both AMPK-dependent and AMPK-independent manners. In addition, it is obvious that glucose-lowering effects are involved in the renoprotective activity of metformin. The renoprotective effects of metformin are shown in Figure 2. The major results of animal studies are summarized in Table 1. Metformin at 300 mg/kg/day in animal studies is considered to be equivalent to the dose for clinical use in human patients (1200–2400 mg/day for a 50–100 kg human patient), normalized by the body surface area [80].

## 5. Clinical Studies

The United Kingdom Prospective Diabetes Study (UKPDS) is the first large-scale randomized clinical trial to demonstrate the effectiveness of intensive glucose reduction and metformin use on diabetic complications in T2D [4,81]. In UKPDS34, metformin use showed a risk reduction of 32% for any diabetes-related endpoint, 42% for diabetes-related death and 36% for all-cause mortality in newly diagnosed overweight T2D individuals [4]. Accordingly, in UKPDS80 (a post-interventional 10-year follow-up of the UKPDS), intensive therapy with metformin resulted in a risk reduction of 33% in myocardial infarction, 20% in stroke and 16% in microvascular complications, defined as vitreous hemorrhaging, retinal photocoagulation, or renal failure in T2D patients [5]. 

In a short-term study, the effect of switching from glibenclamide (sulfonyl urea: SU) to metformin on microalbuminuria in T2D patients was examined [82]. In that study, a total of 51 T2D patients were allocated to the glibenclamide or metformin group and followed for 12 weeks. At the end of the study, metformin had significantly reduced the urine albumin secretion by a mean of 24.2 mg/day [82]. 

Another study investigating the short-term (16 weeks) or long-term (4.3 years) effects of combination of metformin with insulin therapy failed to demonstrate the superiority of metformin’s effect on urinary albumin secretion in T2D individuals, although metformin did improve the endothelial function [83,84]. However, an analysis of a long-term study showed that metformin use significantly reduced the risk of macrovascular complications by 39%, which may have been due in part to body weight loss [85]. 

In A Diabetes Outcomes Prevention Trial (ADOPT), a total of 4351 drug-naïve T2D patients were randomly allocated to monotherapy of metformin or rosiglitazone (PPARγ agonist) or glyburide (SU) and followed for five years [86]. At the end of the study, metformin use showed the highest increment in the albumin-to-creatinine ratio (ACR) relative to other comparators (changes from baseline: +20.9% for metformin, +2.1% for rosiglitazone and +6.1% for glyburide). Changes from baseline in eGFR were +1.4% for metformin, +5.1% for rosiglitazone and –0.4% for glyburide, respectively [86]. 

Recently, a retrospective cohort study including 10,426 T2D patients with CKD stage 3 (eGFR 30–45 mL/min/1.73 m^2^) demonstrated that long-term metformin use was associated with 35% (hazard ratio (HR) 0.65; 95% confidence interval (CI) (0.57–0.73) and 33% (HR 0.67; 95% CI 0.58–0.77) risk reductions in all-cause mortality and ESRD progression, respectively (median follow-up period: 7.3 ± 4.8 years) [87]. 

## 6. Metformin Use in Patients with an Impaired Renal Function

Metformin should be carefully administered to patients with CKD, as it can increase the risk of lactic acidosis. The precise mechanisms underlying metformin-associated lactic acidosis (MALA) remain unknown. MALA in CKD is considered to be associated with metformin’s pharmacokinetics. Metformin is filtered from the glomerulus and secreted from proximal tubules in a non-metabolized form [88]. Therefore, under conditions of an impaired renal function, metformin accumulates and impairs the mitochondrial function, oxygen consumption and hepatic gluconeogenesis using lactate, leading to the accumulation of lactate and MALA [88]. 

In particular, metformin should not be prescribed for patients with advanced CKD, due to an increased mortality risk associated with metformin use in those patients [89]. However, a systemic review by Inzucchi et al. documented that the serum metformin levels generally remained within the therapeutic range, and the lactate concentrations were not substantially increased when used in patients with mild to moderate CKD (eGFR 30–60 mL/min/1.73 m^2^) [90]. Therefore, it is now widely accepted that metformin can be prescribed to patients with an eGFR ≥ 30 mL/min/1.73 m^2^ after adjusting the dose depending on the renal function.

The beneficial use of metformin for treating moderate CKD has been reported. A study that investigated the relationship between metformin use and mortality among T2D patients with atherothrombosis demonstrated a 36% risk reduction of mortality in subjects with eGFR 30–60 mL/min/1.73 m^2^ (HR 0.64; 95% CI, 0.48–0.86) [91]. An analysis from the Swedish National Diabetes Register (4 year of mean follow-up period) showed that metformin use reduced the all-cause mortality (HR 0.87; 95% CI, 0.77–0.99) in patients with eGFR 45–60 mL/min/1.73 m^2^ [92]. Consistent with these observations, another cohort study demonstrated that metformin use was associated with a risk reduction of mortality compared with SU use in patients across all ranges of eGFR, including CKD stage 3 [93]. A recent study found that metformin use was associated with a reduced risk of kidney disease composite outcome, defined as ESRD or death (HR 0.77; 95%CI, 0.61–0.98), in patients with CKD stage ≥ 4 compared with non-users. In that study, metformin use was also associated with all-cause mortality (HR 0.49; 95% CI 0.36–0.69) and cardiovascular death (HR 0.49; 95% CI 0.32–0.74) [94]. 

The maximum dose of metformin in CKD is recommended to be 2550 mg in stage 1 and 2 (eGFR > 60 mL/min/1.73 m^2^), 1500 mg in stage 3A (eGFR 45–60 mL/min/1.73 m^2^) and 1000 mg in stage 3B (eGFR 30–45 mL/min/1.73 m^2^). In stages 4 and 5, metformin use is considered to be contraindicated [90]. Lalau et al. reported that excessive metformin concentrations and lactate levels were not observed when metformin was administered at 1500 mg in CKD stage 3A, 1000 mg in CKD stage 3B, or 500 mg in CKD stage 4 after 4 months’ follow-up [95]. However, metformin should be administered with caution in cases with an eGFR < 30 mL/min/1.73 m^2^.

Taken together, these findings suggest that metformin use may help suppress ESRD progression and CVD among patients with CKD stage 3. A summary of clinical effects of metformin in DKD is shown in Table 2. 

## 7. Conclusions and Perspectives

Metformin is the preferred therapeutic option for T2D. A series of experimental studies revealed that metformin has beneficial effects on DKD with its ability to attenuate inflammation, oxidative stress and fibrosis. These renoprotective effects of metformin are potent, at least in animal models. In clinical settings, the effectiveness of metformin on DKD is modest. The reason for these observations remains unknown but it may also be related to the fact that albuminuria was used as renal outcome in most studies. In animal models, metformin has shown prominent inhibitory effects on tubulo-interstitial fibrosis in both diabetic and non-diabetic models. As albuminuria mainly reflects glomerular lesion, focusing on renal fibrosis may provide different results. The long-term benefits of metformin on ESRD and CVD in patients with moderate CKD have emerged. As described previously, heterogeneity of DKD has emerged. Clarifying the efficacy of metformin for a non-albuminuric DKD phenotype, including aging-related renal dysfunction, will be interesting. Theoretically, metformin use may aid in relieving these conditions. The administration of metformin in advanced CKD patients should be discontinued to prevent MALA. In addition, metformin use in elderly individuals should performed with care in order to avoid MALA. Further studies will be required to determine the appropriate metformin dose for elderly T2D patients.

Because DKD involves multiple mechanisms, combination therapy may be ideal to prevent DKD progression. Metformin can be useful as baseline therapy, but which class of drug is most effective for combination administration is unclear. Recently, SGLT2 inhibitors have been in the spotlight because these drugs have been shown to exert renoprotective effects independent of their glucose-lowering effects. For example, in the EMPA-REG OUTCOME (empagliflozin) and CAVNVAS (canagliflozin), approximately 60% of participants with eGFR < 60 mL/min/1.73 m^2^ and 80% of those with eGFR ≥ 60 mL/min/1.73 m^2^ were taking metformin [96,97]. However, the cardiovascular and renal benefits of empagliflozin were observed irrespective of the baseline glucose-lowering therapies in the EMPA-REG OUTCOME. Of note, there was a greater reduction in the risk of DKD for metformin non-users (HR 0.47; 95% CI, 0.37–0.59) than for metformin users (HR 0.68; 95% CI, 0.58–0.79) [98]. LEADER (liraglutide) [58], SUSTAIN-6 (semaglutide) [59] and REWIND (dulaglutide) [60] have demonstrated renoprotective effects of GLP-1RAs. In these trials, close to 80% of participants were taking metformin at baseline. It remains unclear how metformin affects these results. Further studies will be required to clarify how metformin can be used effectively in combination with other glucose-lowering agents. Interestingly, SGLT2 inhibitors have been shown to enhance the AMPK and Sirt1 signaling pathway. It is proposed that metformin primarily acts through the activation of AMPK and that SGLT2 inhibitors act principally through an enhanced SIRT1 signaling pathway [99,100,101,102]. Whether or not metformin and SGLT2 inhibitors stimulate these pathways synergistically remains unclear. Further studies will be needed to elucidate the mechanisms concerning how those drugs act together in common renoprotective pathways.

Unfortunately, metformin should be discontinued in cases of advanced CKD despite its favorable effects. To maximize the renoprotective benefits of metformin, it may be necessary to use it in combination with SGLT2 inhibitors or incretin-based therapies from the early stage of DKD. 

## Figures and Tables

**Table 1:** Results of animal studies describing the renoprotective effects of metformin. The administration of metformin can reduce renal inflammation, oxidative stress and fibrosis under diabetic and non-diabetic conditions. STZ: streptozotocin, TGF-β: transforming growth factor β, db/db: C57BL/KsJ-Lepr^db^/Lepr^db^, GLP-1R: glucagon-like peptide 1 receptor, ZDF: Zucker diabetic fatty, UUO: unilateral urethral obstruction, GBM: glomerular basement membrane, NCC: Na-Cl cotransporter.

**Studies**	**Models**	**Metformin Dose**	**Key Findings**
Zhang et al. 2017 [45]	STZ diabetic rats	70 mg/kg/d 13 weeks	Albuminuria↓ Renal TGF-β ↓ Oxidative stress↓
Kim et al. 2015 [47]	db/db mice	30 mg/kg/d 4 weeks	Glomerular GLP-1R↑
Borges et al. 2020 [49]	Subtotal nephrectomy rats	300 mg/kg/d 120 days	Albuminuria↓ Renal fibrosis↓ Mitochondrial function↑
Polianskyte-Prause et al. 2019 [57]	db/db mice	250 mg/kg/d 12 days	Podocyte apoptosis↓ Insulin signaling↑
Takiyama et al. 2011 [62]	ZDF diabetic rats	500 mg/kg/d 30 weeks	Albuminuria↓ Tubular injury↓ Renal hypoxia↓
Lee et al. 2018 [65]	folic acid nephropathy mice	0.4 mg/L in drinking water 2 weeks	Renal fibrosis↓ Lipid availability↑
Christensen et al. 2019 [69]	STZ diabetic rats	250 mg/kg/d 4 weeks	Medullary tissue oxygen tension↑ Mitochondrial superoxide↓
Jiang et al. 2020 [71]	db/db mice	200 mg/kg/d 16 weeks	Senescence of renal tubular epithelial cells↓
Christensen et al. 2019 [73]	UUO mice (non-diabetic)	500 mg/kg/d 7 days	Immune cell infiltration↓ Tubular damage↓ Renal fibrosis↓ STAT3↓
Feng et al. 2017 [74]	UUO mice (non-diabetic)	200 mg/kg/d 10 days	Renal fibrosis↓ TGF-β/Smad3↓
Xu et al. 2020 [76]	High-fat-diet-induced diabetic rats	150-500 mg/kg/d 8weeks	Autophagy↑ Sirt1/FoxO1↑ GBM thickness↓ Tubular injury↓
Ren et al. 2020 [77]	High-fat diet and low-dose STZ diabetic rats	250 mg/kg/d 8 weeks	Autophagy↑ Sirt1/FoxO1↑ Oxidative Stress↓ Glomerulosclerosis↓
Hashimoto et al. 2018 [78]	non-diabetic mice	300 mg/kg/d 5 days	Urinary sodium excretion↑ NCC activity↓

**Table 2:** Clinical effects of metformin on DKD. Metformin use is associated with reduced mortality in T2D with CKD. However, metformin increases the mortality risk in patients with advanced CKD. The dose of metformin is indicated when such data were available. UKPDS: United Kingdom Prospective Diabetes Study, T2D: type 2 diabetes, RR: relative risk, RCT: randomized controlled trial, DKD: diabetic kidney disease, ESRD: end stage renal disease, CKD: chronic kidney disease, HR: hazard ratio

**Studies**	**Patients**	**Results**
UKPDS80. 2008 [5] Post-trial monitoring (10 years)	Overweight T2D n = 3277	Metformin (2550 mg/day) reduced microvascular complications (RR 0.84 [0.60–1.17])
Amador-Licona et al. 2000 [82] RCT (12 weeks)	T2D with incipient DKD n = 51	Metformin (850 mg/day) reduced urinary albumin excretion by switching from glibenclamide (5 mg/day)
Kooy et al. 2009 [85] RCT (4.3 years)	T2D with insulin therapy n = 390	Metformin (850 mg/day) did not reduce DKD (versus placebo)
Lachin et al. 2011 [86] RCT (5 years)	T2D with drug-naïve n = 4351	No beneficial effects of metformin (2000 mg/day) on DKD compared with rosiglitazone (8 mg/day) and glyburide (15 mg/day)
Kwon et al. 2020 [87] Retrospective, Observational, Cohort Study (7.3 years)	T2D with DKD n = 10,426	Metformin use was associated with lower all-cause mortality (RR 0.65 [0.57–0.73]) and ESRD progression (RR 0.67 [0.58–0.77])
Hung et al. 2015 [89] Retrospective, Observational, Cohort Study (2.1 years)	T2D with advanced CKD n = 12,350	Metformin use was an independent risk factor for mortality (HR 1.35 [1.20–1.51])
Roussel et al. 2010 [91] Observational Study (2 years)	T2D with established atherothrombosis (including CKD) n = 19,691	Metformin use was associated with lower all-cause mortality (RR 0.64 [0.48–0.86]) in patients with eGFR 30–60
Ekstrom et al. 2012 [92] Observational, Cohort Study (4 years)	T2D (including with CKD) n = 51,675	Metformin use reduced all-cause mortality (HR 0.87 [0.77–0.99]) in patients with eGFR 45–60
Marcum et al. 2018 [93] Observational, Cohort Study (5 years)	T2D with monotherapy of metformin or SU (including CKD) n = 175,296	Metformin use was associated with a lower mortality (versus SU) across all ranges of eGFR (HR 0.59–0.80). The greatest risk difference was observed in the eGFR category 30–44
Charytan et al. 2019 [94] Retrospective Study (4 years)	T2D with CKD (stage 3 and higher) n = 591 metformin users and 3447 non-users	Metformin use reduced the risk of all-cause mortality (HR 0.49 [0.36–0.69]), cardiovascular death (HR 0.49 [0.32–0.74), cardiovascular composite (HR 0.67 [0.51–0.88]) and the kidney disease composite (HR 0.77 [0.61–0.98]) (versus non-users)

## Abbreviations

DKD	diabetic kidney disease
CKD	chronic kidney disease
CVD	cardiovascular disease
T2D	type 2 diabetes
TGF-β	transforming growth factor-β
ECM	extracellular matrix
NF-κB	nuclear factor-κB
eGFR	estimated glomerular filtration rate
GLP-1	glucagon-like peptide 1
GDF 15	growth/differentiation factor 15
LKB-1	liver kinase B-1
AMPK	AMP-activated kinase
OCT	organic cationic transporter
MATE	multidrug and toxin extrusion
mTOR	mammalian target of rapamycin
FAO	fatty acid oxidation
UUO	unilateral urethral obstruction
ACC	acetyl-CoA carboxylase
MALA	metformin-associated lactic acidosis
CAVNVAS	Canagliflozin Cardiovascular Assessment Study Program
LEADER	Liraglutide Effect and Action in Diabetes: Evaluation of Cardiovascular Outcome Results
SUSTAIN-6	Trial to Evaluate Cardiovascular and Other Long-term Outcomes with Semaglutide in Subjects with Type 2 Diabetes
REWIND	Researching Cardiovascular Events with a Weekly Incretin in Diabetes
